# Ectopic intranasal canine tooth in a child: A rare case report and literature review

**DOI:** 10.1016/j.ijscr.2019.01.037

**Published:** 2019-01-31

**Authors:** Abdulaziz AlMulhim, Ali AlMomen, Abdulrahman AlKhatib

**Affiliations:** Department of Otolaryngology, King Fahad Specialty Hospital, Dammam, Saudi Arabia

**Keywords:** Nasal tooth, Ectopic tooth, Nasal obstruction, Inferior turbinate

## Abstract

**Introduction:**

Intranasal teeth are a rare form of ectopic teeth. Their clinical manifestation are truly variable. In children, intranasal teeth are typically associated with cleft lip and alveolus.

**Presentation of case:**

Here, we report a case of 11 years –old girl presented with right nasal obstruction and occasional headache without any obvious etiology. 0Computed tomography of the paranasal sinuses (coronal and axial view) revealed displaced right upper maxillary tooth with the crown oriented inferiorly and medially toward and within the lower right anterior nasal cavity. She underwent surgery by anterior rhinoscopy and endoscopic guidance. The patient’s symptoms were resolved completely post-operatively and remained symptom-free for 18 months postoperatively.

**Discussion:**

Ectopic intranasal tooth is a rare phenomenon, with a male predominance and around half of all patients are diagnosed before adulthood. Ectopic intranasal tooth arising from inferior turbinate is very rare. No clear etiological factor has been suggested in most of the reported cases. Idiopathic etiology has been described as an etiologic factor for ectopic teeth. The treatment of intranasal teeth is early surgical extraction to alleviate the symptoms and prevent the possible morbidities.

**Conclusion:**

Intranasal teeth are a rare form of ectopic teeth encountered to otolaryngology clinic and may cause a variety of symptoms and complications. CT is very useful; it confirms the diagnosis and facilitates surgical planning. Early diagnosis and treatment are very important to avoid their possible complications.

## Highlights

• Ectopic teeth are commonly seen in palate.
• The pathogenesis of ectopic teeth eruption is not fully recognized.
• In children, intranasal ectopic teeth occur more frequent in patients with cleft lip and alveolus.
• Most cases of the ectopic tooth are asymptomatic and identified incidentally.
• The management of the ectopic teeth is tooth extraction.

## 1 Introduction

Ectopic intranasal teeth are rare and can occur in different locations. The incidence of nasal teeth ranges from 0.1 to 1% of the population. Ectopic teeth are commonly seen in palate, maxillary sinus and the floor of the nasal cavity respectively and rarely been reported to be on the inferior turbinate. In children, intranasal ectopic teeth occur more frequent in patients with cleft lip and alveolus [1,2].

The pathogenesis of ectopic teeth eruption is not fully recognized but it may occur due to genetic factors, cleft palate, odontogenic infections, trauma, crowding of displacement due to cyst or tumors [3,4]. Most cases of the ectopic tooth are asymptomatic and identified incidentally on routine clinical and radiological examination [5]. It is important to identify it as it may cause serious morbidity. The standard management of the ectopic teeth is tooth extraction. In this article, we report an unusual and rare case of an intranasal ectopic tooth in a child without any obvious etiology.

This case has been reported in line with the SCARE criteria [6].

## 2 Presentation of case

**Fig. 1:**
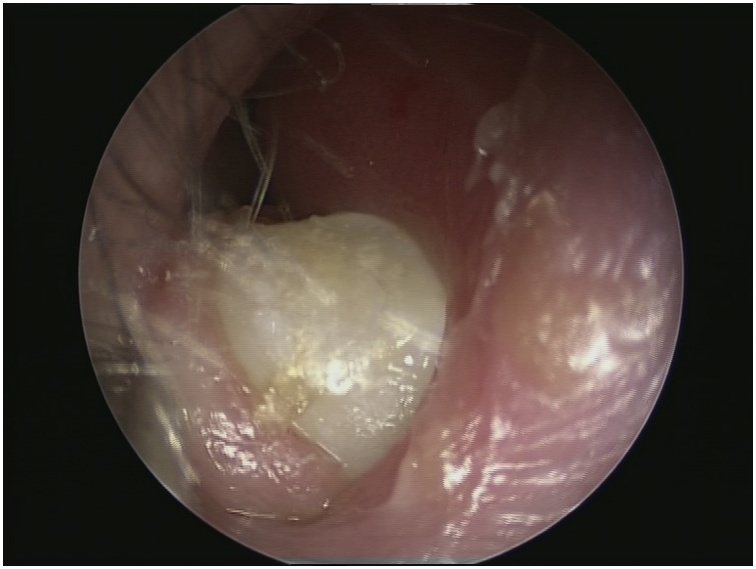
Hard bony mass filling the right nasal cavity.

An 11 years-old girl a known case of acute lymphoblastic leukemia on remission that has received her last chemotherapy 4 months back has been referred to otolaryngology clinic with a history of progressive right-sided nasal obstruction along with occasional headache. This complaint has developed over a period of 1 year prior to her presentation. On examination, she has a collapsed right lower lateral cartilage, supratip depression, and hard bony mass filling the right nasal cavity (from the anterior aspect of inferior turbinate touching the nasal septum) (Fig. 1). Her intraoral dentition was normal with no cleft palate or other congenital anomalies. No previous history of maxillofacial trauma, surgery or nasal foreign body was elicited.

**Fig. 2:**
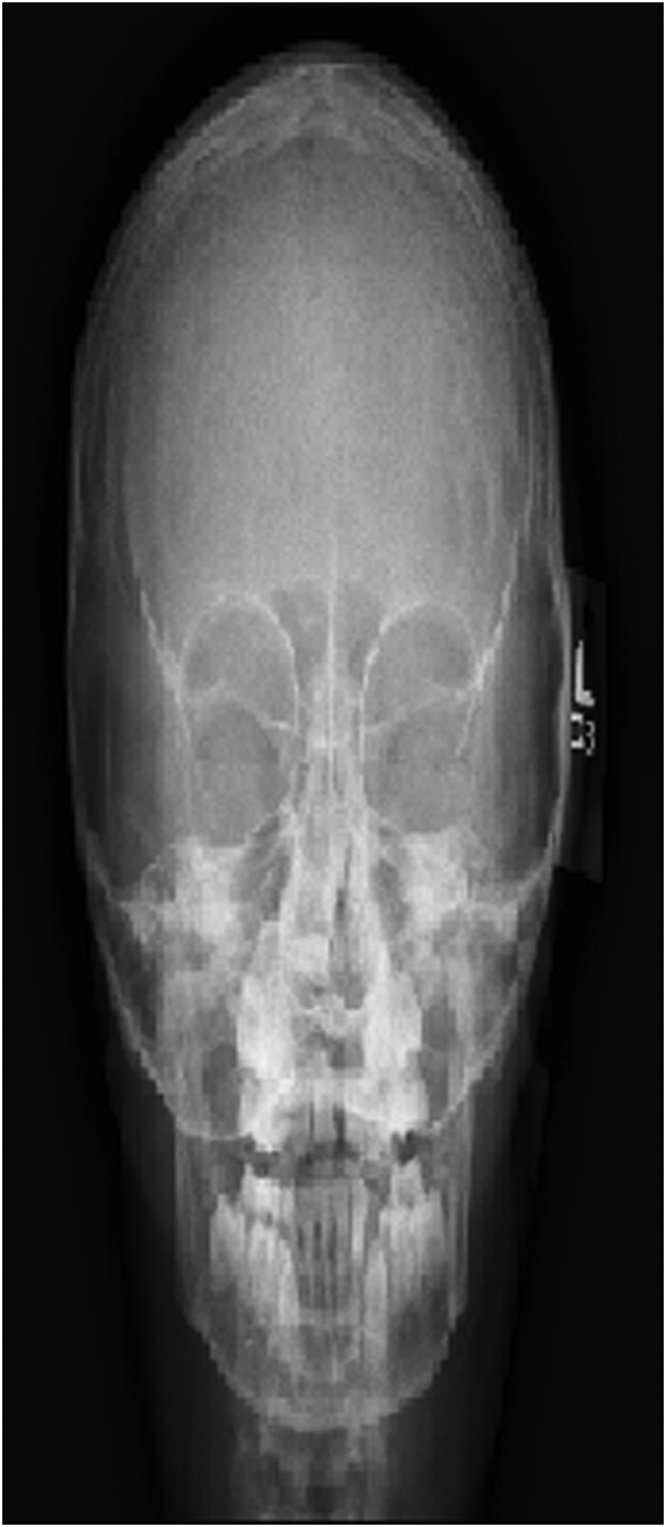
Plain x-ray (AP view) of the skull revealing radiopaque mass filling the right nasal cavity.

**Fig. 3:**
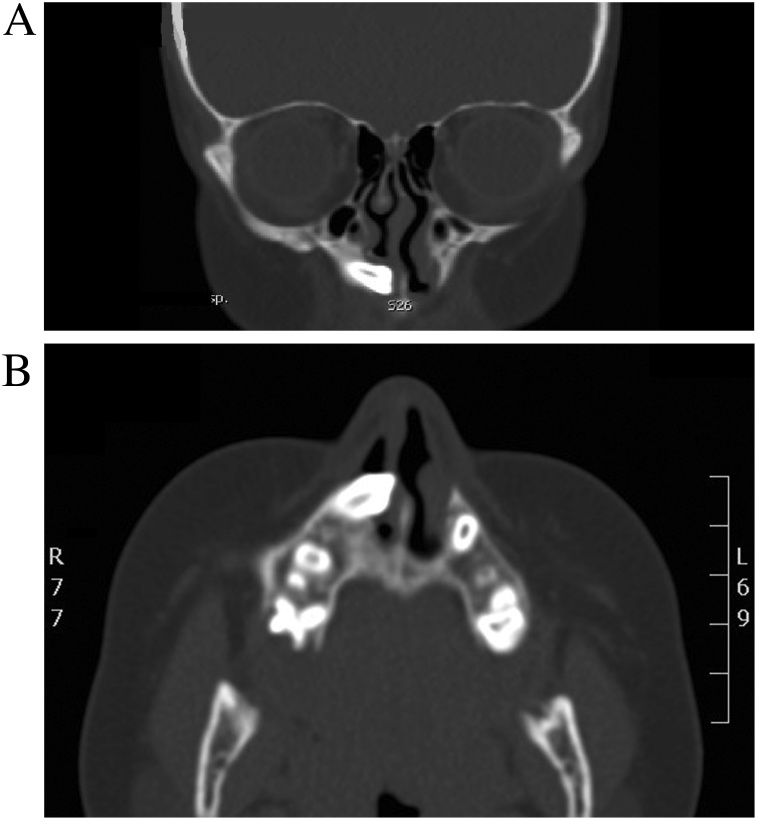
A,B: Computed tomography of the paranasal sinuses (coronal and axial view) revealing displaced right upper maxillary tooth with the crown oriented inferiorly and medially toward and within the lower right anterior nasal cavity.

Plain x-ray (AP view) of the skull revealed radiopaque mass filling the right nasal cavity (Fig. 2). Computed tomography of the paranasal sinuses (coronal and axial view) revealed displaced right upper maxillary tooth with the crown oriented inferiorly and medially toward and within the lower right anterior nasal cavity (Fig. 3A, B) with no destruction of the adjacent structures.

**Fig. 4:**
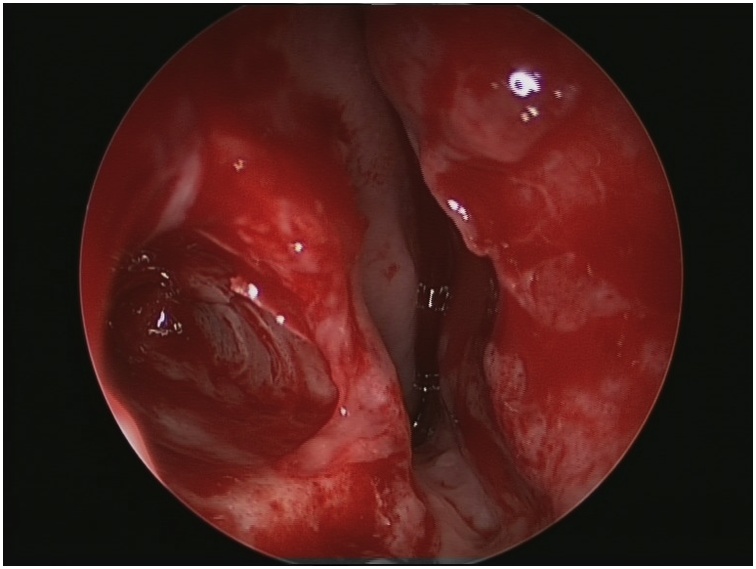
Post Operative.

**Fig. 5:**
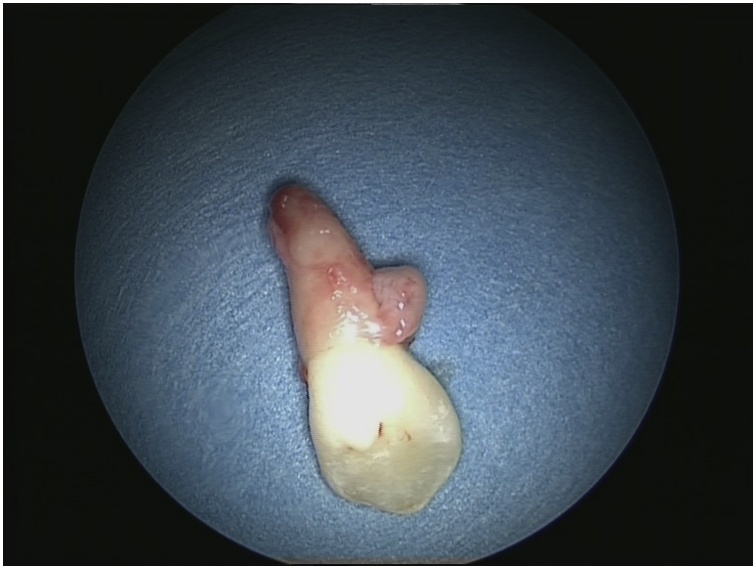
Gross specimen showing a canine tooth.

Informed consent was obtained from her parents she underwent surgery by anterior rhinoscopy and endoscopic guidance, intranasal mass was found and has been removed by forceps without any complications (Fig. 4). The specimen was sent for histopathologic examination that confirmed the diagnosis of intranasal ectopic tooth. The gross specimen showed a canine tooth (Fig. 5). The patient’s symptoms were resolved completely post-operatively and remained symptom-free for 18 months postoperatively.

## 3 Discussion

Ectopic intranasal tooth is a rare phenomenon, with a male predominance and around half of all patients are diagnosed before adulthood [7]. Ectopic intranasal tooth arising from inferior turbinate is very rare, from 1966 to 2014 ectopic tooth arising from inferior turbinate in two adult female skulls has been reported by Ray [8] and in a pediatric patient by Yu [9]. No clear etiological factor has been suggested in most of the reported cases. However, a number of etiological causes were raised, such as dental trauma, maxillary osteomyelitis, and development of a defect such as cleft palate, nasal infections and genetic factors [9,10]. In children, intranasal ectopic teeth occur more frequent in patients with cleft lip and alveolus [1,2].

The potential of chemotherapeutic effects in five of 23 patients who received treatment for tumors located outside the head and neck region included acquired amelogenesis imperfecta, microdontia of bicuspid teeth, and a tendency toward thinning of roots with an enlarged pulp chamber [11]. In this case, the patient was diagnosed with acute lymphoblastic leukemia and was treated with chemotherapy for 2 years and this factor may predispose to supernumerary teeth. However, idiopathic etiology has been described as an etiologic factor for ectopic teeth [7]. In most cases, intranasal ectopic teeth are most commonly seen as a single unilateral tooth rather than multiple teeth in both nasal cavities [1,9]. The diagnosis of an intranasal tooth is not difficult but it lacks clinical symptoms and has a vague clinical presentation that led it to be easily missed. Patients usually are asymptomatic but may present with different symptoms, such as facial pain, nasal obstruction, headache, epistaxis, foul-smelling rhinorrhea, external nasal deformities, and nasolacrimal duct obstruction [12]. Clinically, ectopic intranasal teeth may present as hard white masses not surrounded by a nasal mucosa, for which diagnosis is usually straightforward. Nevertheless, sometimes, the tooth may be embedded in the nasal mucosa and surrounded by debris, granulation tissue, and ulcerative materials, for which a differential diagnosis should be formulated [13] such as a foreign body, rhinolith, tumor, osteoma odontoma or a cyst lesion [14,15]. Radiographically, the nasal teeth in our patients appeared as dense radiopaque shadow with the same attenuation as that of the oral teeth. Radiographic studies help to differentiate between these possibilities and in particularly computed tomography is a very useful in means that it allows to confirm the diagnosis and facilitate surgical planning [7,14]. Mainly the diagnosis of intranasal teeth is made based on clinical, radiographic findings.

The treatment of intranasal teeth is early surgical extraction to alleviate the symptoms and prevent the possible morbidities that includes rhinosinusitis, osteomyelitis, dacryocystitis, nasal septal abscess or perforation, oronasal or intraoral fistula, aspergillosis, and nasal deformity [12,[14], [15], [16]]. The extraction of intranasal teeth can be performed by anterior rhinoscope under endoscopic guidance for clear visualization to minimize injury to nearby structures.

## 4 Conclusion

Intranasal teeth are a rare form of ectopic teeth encountered to otolaryngology clinic and may cause a variety of symptoms and complications. Their diagnosis is not difficult; it depends mainly on its characteristic clinical and radiological findings. In most cases, the cause of intranasal tooth remains unclear. CT is very useful; it confirms the diagnosis and facilitates surgical planning. Early diagnosis and treatment are very important to avoid their complications.

## Conflicts of interest

Nothing to declare.

## Funding

No sources of funding.

## Ethical approval

Case report is exempt from ethical approval in our institution.

## Consent

Written informed consent was obtained from the patient parents for publication of this case report.

## Author contribution

Abdulaziz AlMulhim - Data collection, Editing of manuscript.

Ali AlMomen – Data collection, Editing of the manuscript.

Abdulrahman AlKhatib – Writing the original draft.

## Research Registration Number

NA.

## Guarantor

Abdulaziz AlMulhim.

## Provenance and peer review

Not commissioned externally peer reviewed.
